# Insights into the Anticancer Mechanisms Modulated by Gamma and Delta Tocotrienols in Colorectal Cancers

**DOI:** 10.1093/nutrit/nuae108

**Published:** 2024-08-24

**Authors:** Ali Qusay Khalid, Tabarek Najeeb Zaidan, Saatheeyavaane Bhuvanendran, Kasthuri B Magalingam, Shaza M Mohamedahmed, Premdass Ramdas, Ammu K Radhakrishnan

**Affiliations:** Food as Medicine Research Strength, Jeffrey Cheah School of Medicine and Health Sciences, Monash University Malaysia, 47500 Bandar Sunway, Malaysia; Department of Food Science and Nutrition, Faculty of Applied Sciences, UCSI University, UCSI Heights, Cheras, 56000 Kuala Lumpur, Malaysia; Food as Medicine Research Strength, Jeffrey Cheah School of Medicine and Health Sciences, Monash University Malaysia, 47500 Bandar Sunway, Malaysia; Food as Medicine Research Strength, Jeffrey Cheah School of Medicine and Health Sciences, Monash University Malaysia, 47500 Bandar Sunway, Malaysia; Food as Medicine Research Strength, Jeffrey Cheah School of Medicine and Health Sciences, Monash University Malaysia, 47500 Bandar Sunway, Malaysia; Food as Medicine Research Strength, Jeffrey Cheah School of Medicine and Health Sciences, Monash University Malaysia, 47500 Bandar Sunway, Malaysia; Food as Medicine Research Strength, Jeffrey Cheah School of Medicine and Health Sciences, Monash University Malaysia, 47500 Bandar Sunway, Malaysia

**Keywords:** tocotrienols (T3s), vitamin E, colon cancer, colorectal cancer (CRC), anti-proliferation, apoptosis, cell cycle, telomerase, metastasis, synergism, immunity

## Abstract

Colorectal cancer (CRC) is a growing concern all over the world. There has been a concerted effort to identify natural bioactive compounds that can be used to prevent or overcome this condition. Tocotrienols (T3s) are a naturally occurring form of vitamin E known for various therapeutic effects, such as anticancer, antioxidant, neuroprotective, and anti-inflammatory activities. The literature evidence suggests that two T3 analogues, ie, gamma (γ)- and delta (δ)-T3, can modulate cancers via several cancer-related signaling pathways. The aim of this review was to compile and analyze the existing literature on the diverse anticancer mechanisms of γT3 and δT3 exhibited in CRC cells, to showcase the anticancer potential of T3s. Medline was searched for research articles on anticancer effects of γT3 and δT3 in CRC published in the past 2 decades. A total of 38 articles (26 cell-based, 9 animal studies, 2 randomized clinical trials, and 1 scoping review) that report anticancer effects of γT3 and δT3 in CRC were identified. The findings reported in those articles indicate that γT3 and δT3 inhibit the proliferation of CRC cells, induce cell cycle arrest and apoptosis, suppress metastasis, and produce synergistic anticancer effects when combined with well-established anticancer agents. There is preliminary evidence that shows that T3s affect telomerase functions and support anticancer immune responses. γT3 and δT3 have the potential for development as anticancer agents.

## INTRODUCTION

**Figure 1.**
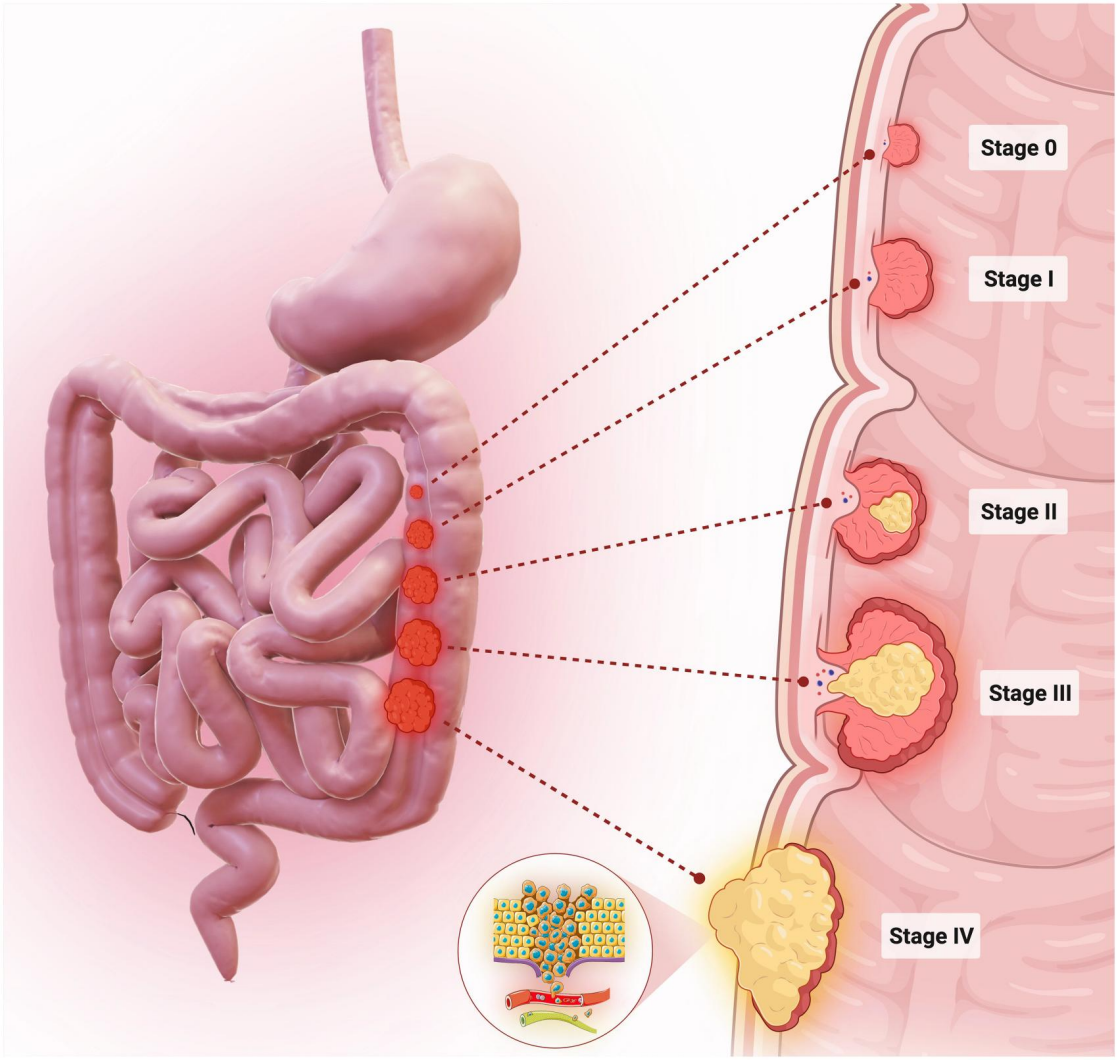
Stages of Human Colorectal Cancer. The initial phase of colorectal malignancies is referred to as Stage 0, which denotes a highly nascent form of cancer. Stage 0 can progress to Stage I to IV with time (4). Stage I denotes a lesser extent of cancer metastasis, while Stage IV indicates a more extensive dissemination of cancer Abbreviation: δT3, δ-Tocotrienol

One of the leading causes of death worldwide is cancer.^1^ There are currently more than 100 forms of cancers linked to various human organs, and the most frequently diagnosed cancer worldwide is colorectal cancer (CRC).^2^ Although the incidence of CRC is increasing worldwide, its prevalence appears to differ in different countries.^1–3^ It is estimated that, by the year 2040, the number of new CRC cases worldwide will hit 3.2 million.^4^ The CRC may occur in various parts of the human colon (Figure 1). The most prevalent form of CRC is adenocarcinoma.^5^ Other types of CRC include carcinoid tumors,^6^ gastrointestinal stromal tumors,^7^ colorectal lymphomas,^8^ and hereditary CRC, which can affect individuals in different generations of the one family who have or may develop CRC (eg, hereditary non-polyposis colon cancer [HNPCC] and familial adenomatous polyposis [FAP]).^9^

### Risk factors for colorectal cancer

Risk factors for cancers can be broadly classified as modifiable (environment, lifestyle, nutrition, supplements, drugs) or nonmodifiable (genetics, age) factors. The same applies to CRC. In the literature, the development of CRC is reported to be influenced by environment, diet, lifestyle (obesity, physical activity, alcohol, smoking), gut microbiota, medications, and other factors.^10–12^ Recently, Inamura et al^11^ described cancer as a microenvironmental systemic and environmental disease in which modifiable and nonmodifiable risk factors can influence tumor phenotypes by modulating systemic conditions and the tumor microenvironment (TME). Exposure to environmental toxicants, such as asbestos, industrial complex pollutants, and pesticides,^13^ as well as high consumption of processed and red meats,^14^ and high fat intake^14^ have been associated with an increased risk of CRC. Lifestyle factors such as obesity, physical inactivity, alcohol drinking, and smoking can also affect treatment efficacy and overall survival.^11^ According to Ogino et al,^12^ prudent lifestyle habits can be correlated with a reduced incidence of CRC that does not vary according to molecular subtypes or anatomic subsites. Increased incidence of early-onset CRC has been reported, which has led to an increase in CRC-related deaths in patients under 50 years of age.^15^ Some of these risk factors may influence various carcinogenic processes to varying extents, such as modifying the genome, epigenome, transcriptome, metagenome, or other intrinsic factors causing normal cells to acquire neoplastic characteristics.^12^^,^^16^

Around 10% of CRC cases are attributed to germ-line mutations, while the overwhelming majority result from an initiating exposure event that transpired decades before the diagnosis.^17^ Mutations in driver genes such as adenomatous polyposis coli (*APC*), tumor-suppressor protein 53 (*TP53*), Kirsten rat sarcoma virus (*KRAS*), phosphatidylinositol-4,5-bisphosphate 3-kinase catalytic subunit-alpha (*PIK3CA*), and B-Raf proto-oncogene, serine/threonine kinase (*BRAF*) have been implicated in the development of CRC.^16^^,^^18^

## TREATMENT OF COLORECTAL CANCER

At present, several chemotherapeutic drugs are being used to treat CRC. It has been reported that CRC patients tend to develop resistance to the drugs used in chemotherapy over time, which results in poor prognosis.^19–21^ Hence, researchers and doctors seek additional therapeutic agents, ideally with fewer side effects, to treat these patients. Several factors appear to plague the development of new anticancer agents, such as poor bioavailability,^22^ lack of solubility in aqueous solutions,^23^ and the side effects these drugs can cause in cancer patients.^24^^,^^25^ Bioactive food components demonstrate significant promise as a scientifically sound strategy for preventing and potentially treating CRC. Numerous natural products, collectively identified as “phytochemicals” or “nutraceuticals,” have exhibited noteworthy anticancer properties to CRC. Among these, the tocotrienols (T3s), a less familiar form of vitamin E, stand out as compelling bioactive compounds. Scientific evidence supports their potential efficacy in countering CRC, reinforcing the viability of incorporating such bioactive compounds into preventive and therapeutic approaches.^26^

## TOCOTRIENOLS

Vitamin E is a fat-soluble micronutrient that is present in several edible oils, such as palm, annatto bean, and rice bran oils.^27^ There are two naturally occurring forms of vitamin E, ie, tocopherols (Tocs) and T3s. Both forms of vitamin E exist naturally as 4 isoforms, namely, alpha- (α), beta- (β), delta- (δ), and gamma- (γ).^28^ The T3s and Tocs exhibit comparable chemical structures, characterized by a chromanol ring and a 16-carbon phytyl-like tail situated at the C2 position, but the side chain of Tocs is saturated, whereas the side chain of T3s contains 3 double bonds.^29^ Numerous studies have demonstrated the antioxidant properties of vitamin E,^30^ as well as its cardioprotective,^31^ neuroprotective,^32^ and anti-diabetic effects.^33^^,^^34^ The T3s are reported to be 1600 times more potent as an antioxidant than Tocs.^35^^,^^36^ More recent studies show that γT3 and δT3 are promising phytochemicals with anticancer properties,^37–39^ and these compounds exert their anticancer effects by targeting multiple cell signaling pathways,^40^ all of which can be linked to carcinogenesis of CRC and other cancers.^40^^,^^41^ Despite the growing literature supporting the superior health-enhancing effects of T3s compared with Tocs, T3s are yet to be acknowledged as an essential vitamin E. At present, only α-Toc is recognized as an essential form of vitamin E.^42^

Numerous publications document the anticancer properties of T3s,^37–39^ but there remains a scarcity of data concerning the plasma concentrations of T3s in both animal models of CRC and clinical trials. This deficiency in information hampers the ability to substantiate the observed health benefits attributed to these natural compounds. The data on the biodistribution of T3s conducted in healthy mice showed that dietary T3s accumulate in vital organs of the body,^43^ but there is insufficient data from clinical studies. To date, only αToc has been studied in the context of CRC prevention, with the Cancer Prevention Study II Nutrition Cohort showing an inverse association between αToc and CRC risk in women.^44^

In addition, research into the biodistribution of T3s in cancer models is also limited. For instance, in mice induced with hepatoma, γT3 and δT3 were detected in the tumors but not in normal tissues.^45^ Consequently, a pressing need exists for biodistribution studies within CRC models to elucidate the specific locations of accumulation of T3s.

## METHOD

To explore the anticancer effects of γT3 and δT3 in CRC, a comprehensive literature search was conducted of the Medline database, focusing on research articles published between 2003 and 2023. A combination of relevant keywords such as “γ-tocotrienol,” “gamma-tocotrienol,” “δ-tocotrienol,” “delta-tocotrienol,” “colorectal cancer,” “colon cancer,” “anti-cancer,” and “anticancer” was used to perform this search. The aim of the search was to include original research articles that specifically addressed the therapeutic potential, molecular mechanisms, and clinical outcomes associated with these compounds. In the initial selection process, the title and abstract of all articles identified in the search was performed. Following this, a full-text review of each shortlisted paper was undertaken to extract data, including the CRC cell line tested, the dose or the half maximal inhibitory concentration (IC_50_), and the underlying biological pathways/mechanism affected following exposure to γT3 and/or δT3.

This review paper aimed to discuss the anticancer cancer mechanisms of two isoforms of T3s, namely gamma (γ) and delta (δ), in CRC, by reviewing research articles in cell-based in vitro, in vivo, *in papyri*, and preclinical settings published in the past 2 decades on this topic (Table 1^41^^,^^46–77^).

**Table 1.** Selected Studies of the Impact of γT3 and δT3 on Human Colorectal Cancer

Reference	Description of the CRC cell line or tumor	T3 isoforms tested	The half maximal inhibitory concentration (IC_50_)/dose	Mechanism of action
**IN VITRO (CELL-BASED)**				
Szulczewska-Remi et al (2020)^46^	• Caco-2 human colorectal adenocarcinoma cells	γT3, δT3	**γT3**: 41.4 μM **δT3**: 16.8 μM	• Both γT3 or δT3 exhibited anti-proliferation effect against Caco-2.
Husain et al (2019)^47^	• HCT116 and HT29 human CRC cells • NCM460 human colon mucosal cells • SW620 colon adenocarcinoma cells	δT3	50–60 μM	• Inhibited malignant transformation, cell migration, invasion, and markers for epithelial (E-cadherin) to mesenchymal (vimentin) transition, metastasis (matrix metalloproteinase 9), angiogenesis VEGF, inflammation (NF-κB), and Wnt signaling (*β-catenin*). • Induced apoptosis in CRC but not normal colon cells. • δT3 had minimal impact on NCM460, which are healthy colon mucosal cells.
Yusof et al (2019)^48^	• SW837 human CRC cells	γT3 ± 6G	NA	• Inactivated cell cycle process through downregulation of FOXM1 and Wnt signaling pathway and upregulating ATF6, DDIT3, GADD34, CDK1, and p21. • Induced apoptosis by caspase-independent programmed cell death through mitochondrial dysfunction, activation of endoplasmic reticulum-unfolded protein response, and disruption of DNA repair.
Balagoni et al (2017)^49^	• HCT116, Caco-2, SW480, and DLD-1 human CRC cells	γT3 ± MTF	**HCT116** [**γT3**: 26.03 μM] **Caco-2** [**γT3**: 29.5 μM] **SW480** [**γT3**: 39.7 μM] **DLD-1** [**γT3**: 55.9 μM]	• γT3 and MTF showed variable cytotoxicity in different cell lines. • Synergy was observed in HCT116 cells, but no synergy was observed in Caco-2, SW480, or DLD-1 cells.
Wada et al (2017)^50^	• HT29 human colon carcinoma cells	δT3	20.6 μM	• δT3 exerted a direct anti-proliferative effect on HT29.
Abubakar et al (2016)^51^	• HT29 human colon carcinoma cells	δT3 ± JB	**δT3**: 14.39 μM	• Activated caspase-3, -8, and -9 and induced cell death.
Eitsuka et al (2016)^52^	• DLD-1 human colorectal adenocarcinoma cells	δT3 ± FA	**δT3**: ≥10 μM	• Downregulated the expression of human telomerase reverse transcriptase (hTERT). • Ferulic acid improved the bioavailability of δT3.
Jang et al (2016)^53^	• HCT116, HT29, and Caco-2 human colon cancer cells	γT3, δT3, δT-13'-COOH	**(COX-2)**: **δT-13'-COOH**: 4 ± 1 μM; **γT3**: >50 μM; **δT3**: >50 μM	• Inhibited the proliferation of CRC cells, and induced apoptosis/autophagy in CRC cells.
			**(5-LOX)**: **δT-13'-COOH**: 2 ± 0.5 μM; **γT3**: >50 μM; **δT3**: >50 μM	
Prasad et al (2016)^54^	• HT29, HCT116, and Caco-2 human colorectal adenocarcinoma cells	γT3 ± CAP	NA	• Inhibited proliferation of CRC cells with wild-type or mutated *KRAS*.
Abubakar et al (2015)^55^	• HT29 human colon carcinoma cells	γT3 or δT3 ± *Ficus* species–derived alkaloids	**γT3** [14.95 ± 3 µM] **δT3** [14.39 ± 3 µM]	• Synergistic anti-proliferative effects with 2.2 to 34.7-fold reduction of IC_50_ values.
Gu et al (2015)^56^	• HCT116 human colon cancer cells	γT3	NA	• Inhibited spherical cell growth.
Shibata et al (2015)^57^	• DLD-1 human colorectal adenocarcinoma cells	δT3	NA	• Induced cell cycle arrest and apoptosis under hypoxic more than under normoxic conditions. • Upregulated cyclin-dependent kinase inhibitors (p21 and p27). • Activated caspases and suppressed phosphorylation of protein kinase B (Akt) at Thr (308) and Ser (473).
Yusof et al (2015)^58^	• HT29 and SW837 human CRC cells	γT3 ± 6G	**HT29** [255 + 228 µM] **SW837** [170 + 68 µM]	• Increased apoptosis in HT29 (21.2%) and SW837 (55.4%) CRC cells.
Forster et al (2013)^59^	• HT29, Caco-2, and SW480 human colon cancer cells	γT3, δT3	NA	• Suppressed cell growth and modulated tumorigenesis potential.
Zhang et al (2013)^60^	• SW620 and HCT8 human colon carcinoma cells	γT3	**SW620** [31.4 ± 1.51 µM] **HCT8** [32.69 ± 1.29 µM]	• Inhibited the expression level of *β-catenin*, *cyclin D1*, and *c-jun*. • Induced paraptosis-like cell death associated with suppressing Wnt signaling pathway.
Xu et al (2012)^61^	• HT29 human carcinoma cells	γT3	15-60 μM	• Reduced viability. • Induced apoptosis by suppressing the transcriptional activity of β-catenin/Tcf signaling pathway and inhibiting the expression level of *c-myc*, *cyclin D1*, and *survivin*.
Yang et al (2012)^62^	• HCT116 and HT29 human colon carcinoma cells	δT3 γT3 ± ATST ± CXIB	**HCT116** [**γT3**: 17.5 μM] **HT29** [**γT3**: 30 μM]	• The distribution of γT3 and δT3 metabolites were identified in urine and faecal samples as short-chain and double-bond. • The combination of γT3 and ATST greatly potentiated the ability of each individual agent to inhibit cancer cell growth and to induce cell cycle arrest/apoptosis. • The triple combination of γT3, ATST, and celecoxib exhibited synergistic actions when compared with any double combination plus the third agent.
Yue et al (2012)^63^	• SW620 human colon cancer cells	δT3	15.18 μM	• Inhibited proliferation and downregulated the expression levels of *Wnt-1*, *β-catenin*, *c-jun*, and *cyclin D1*.
de Mesquita et al (2011)^64^	• HCT8 human colon cancer cells	δT3 monomer ± δT3 peroxy-dimer	Mixture = 33.28 μM	• The synergistic action reduced cell survival and triggered both apoptosis and necrosis.
Zhang et al (2011)^65^	• SW620 human colon carcinoma cells	δT3	**δT3**=15.2 μM	• Induced paraptosis-like cell death by reducing expression of *β-catenin* and *Wnt-1* by 50%. • Suppressed *cyclin D1*, *c-jun*, and *MMP-7*.
Shibata et al (2010)^66^	• DLD-1 human colorectal adenocarcinoma cells	δT3	NA	• δT3 induced cell cycle arrest and proapoptotic gene/protein expression (eg, *p21*, *p27*, and caspases). • α-Toc not only less cytotoxic to cancer cells, but it also reduces the cytotoxicity of δT3 by inhibiting its cellular uptake.
Yang et al (2010)^67^	• HCT116 and HT29 human colon carcinoma cells	δT3 ± ATST γT3 ± ATST ± CXIB	**HCT116** [**γT3**: 17.5 μM, **δT3**: 20 μM]; **HT29** [**γT3**: 30 μM, **δT3**: 30 μM]	• The combination of δT3 and ATST had a comparable inhibitory effect. • The growth inhibitory effect of ATST was significantly reduced by the presence of mevalonate and geranylgeranyl pyrophosphate, while the effect of γT3 was only slightly decreased. • Farnesyl pyrophosphate and squalene had minimal impact on the inhibitory action of ATST and γT3. • Protein geranylgeranylation, rather than farnesylation, plays a role in the inhibition of colon cancer cell growth.
Shibata et al (2009)^68^	• DLD-1 human colorectal adenocarcinoma cells	δT3	NA	• Suppressed VEGF-induced cell proliferation, migration, tube formation, and adhesion.
Xu et al (2009)^69^	• HT29 human colon carcinoma cells	γT3	NA	• Reduced expression of NF-κB p65 protein and inhibited its nuclear translocation. • Induced cell cycle arrest at G0/G1. • Increased Bax/Bcl-2 ratio. • Activated caspase-3.
Eitsuka et al (2006)^70^	• DLD-1 human colorectal adenocarcinoma cells	γT3, δT3	NA	• The inhibitory potency of the isomer was δT3>γT3. • Increased telomerase inhibition. • Suppressed mRNA expression of *hTERT* and *c-myc* via inhibition of PKC.
**IN VIVO (ANIMAL STUDIES)**				
Yang et al (2021)^71^	• Murine colitis-associated colon cancer	δT3 Metabolite 13'-carboxychromanol	NA	• Inhibited tumorigenesis. • Suppressed pro-inflammatory cytokines. • Modulated gut microbiota.
Husain et al (2019)^47^	• Azoxymethane-induced colorectal carcinogenesis in rats	δT3	NA	• Inhibited colorectal polyps by 70% and CRC by almost 99% compared with the vehicle treatment group. • The cancer inhibition effect of δT3 was more potent than sulindac (50%).
Yang et al (2019)^72^	• Colitis-promoted colon tumorigenesis in mice	δT3, Metabolite 13'-carboxychromanol	NA	• Inhibited TNF-α-induced activation of NF-κB. • Reduced colon tumorigenesis by 36%. • Decreased tumor surface area by 31% (at 0.04% δT3-13'-COOH diet). • Suppressed multiplicity of total polyps by 25% and large polyps by 54%.
Wada et al (2017)^50^	• Colorectal adenocarcinoma mouse embryonic fibroblasts (MEFs) model and Colon 26 cells	δT3	0.4 μM	• δT3 significantly inhibited colorectal adenocarcinoma cell proliferation and suppressed tumor formation. • It affected the tumor stromal environment, suppressing the tumor cell proliferation-promoting COX-2/PGE2 pathway.
Jang et al (2016)^53^	• AOM/DSS-induced colon cancer in a preclinical BALB/c mice model	δT3, Metabolite 13'-carboxychromanol	NA	• Suppressed tumor promotion/multiplicity and inhibited of 5-LOX and COX-2.
Prasad et al (2016)^54^	• Nude mouse xenograft model of human colorectal carcinoma	γT3 ± ATST	NA	• Individual and combined treatments inhibited the expression of *Ki-67*, *cyclin D1*, *MMP-9*, *CXCR4*, *NF-κB*/*p65*, and *VEGF*.
Qureshi et al (2010)^73^	• Macrophage RAW 264 cells	γT3, δT3	NA	• Induced anti-inflammatory effect and TNF-α suppression.
Shibata et al (2008)^74^	• Implanted colorectal adenocarcinoma in nude mice via DLD-1-induced angiogenesis	δT3	NA	• Tumor suppression and dose-dependent inhibition of vessel formation.
Nakagawa et al (2007)^75^	• DLD-1 cells were surgically implanted into an ICR mouse	γT3, δT3	NA	• Suppressed neovascularization of tumor and increased the frequency of avascular zone. • Induced angiogenesis through suppression of FGF-induced cell proliferation, migration, and tube formation.
**CLINICAL TRIAL**				
Vejle Hospital (2022)^76^	• Metastatic CRC	δT3 ± BVZ	In review (Phase II)	• The median time to first hospitalization or death was 3.7 months in the placebo group and NR in the δT3 group, with rare grade 3–4 toxicities except neutropenia. There were no grade 3 or 4 peripheral sensory neuropathy.
Raunkilde et al (2022)^77^	• Unresectable metastatic CRC	δT3 ± FOLFOXIRI	Phase II	• A hazard ratio (HR) of 0.70 for postponing time to Serious Adverse Events (SAEs), but the difference was not statistically significant in early efficacy.
***IN PAPYRI* (SYSTEMATIC OR SCOPING REVIEWS)**				
Khalid et al (2021)^41^	• Human colorectal carcinoma HT29, HCT116, SW620, DLD-1, SW837, SW480, Caco-2, HCT8 cells	γT3, δT3	NA	• Exertion of anticancer effects through 3 major pathways: apoptosis (*BIRC3*, *BIRC5*, *caspase-8*, *caspase-9*, and *PARP1*), transcriptional dysregulation in cancer (*CDKN1A*, *CDKN1B*, *MMP9*, *MYC*, *JUN*, and *RELA*), and cancer progression (*caspase-3*, *CCND1*, *CTNNB1*, *VEGFA*, and *Wnt-1*).

Names of colon cancer cell lines and the T3 isoform tested are shown in bold font.

*Abbreviations:* ATST, atorvastatin, BVZ, bevacizumab; CAP, capecitabine; COX-2, cyclooxygenase-2; CRC, colorectal cancer; CSC, cancer stem cell; CXIB, celecoxib; FA, ferulic acid; 5-FU, 5-fluorouracil; 6G, 6-gingerol; IC_50_, concentration that kills 50% of cultured cells; JB, jerantinine B; PKC, protein kinase C; 5-LOX, 5-lipoxygenase; MTF, metformin; γT3, gamma-T3; δT3, delta-T3; T3s, tocotrienols; NA, not available (not provided in paper).

## ANTI-PROLIFERATIVE EFFECTS

Cell proliferation is a well-defined complex cell division process that is strictly controlled and results in a rise in cell numbers.^78^ If the processes that regulate cell division become aberrant, this would cause abnormal cellular proliferation, which is a hallmark of neoplasia. Hence, performing a quantitative investigation of cell proliferation is crucial to understanding the aggressiveness of cancer cells, which is why cell proliferation studies are often used to evaluate the effects of anticancer and cytotoxic drugs. Growing evidence supports the selective anti-proliferative effects exerted by γT3 and δT3 on cancer cells.^79^^,^^80^ Both γT3 and δT3 exerted strong anti-proliferative effects on various human CRC cell lines such as Caco-2,^46^ HCT116,^47^ SW480,^49^ SW837,^58^ SW620,^60^ HCT8,^64^ DLD-1,^66^ and HT29.^69^ Recently, it was suggested that γT3 and δT3 play a key role in cancers by modulating life-or-death decisions,^81^ and the mechanism may be regulation of the expression of various biomarkers involved in the cell proliferation signaling pathways and limiting of the proliferative ability of the CRC cells.^41^^,^^50^^,^^82^

**Figure 2.**
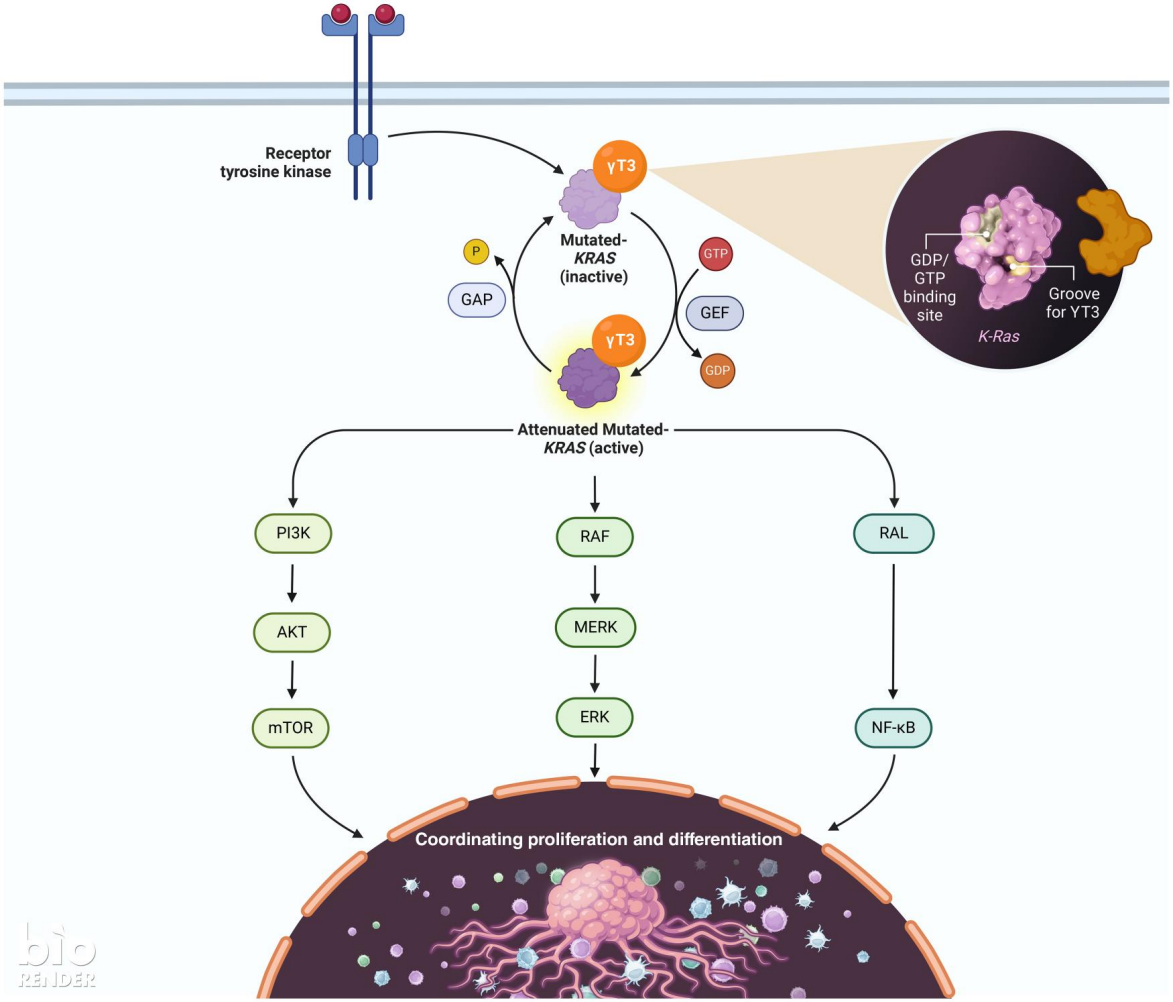
Proposed Mechanisms Through Which γ-tocotrienol May Affect the Proliferation of Colorectal Cancer Cells through the KRAS Pathway in Wild-Type or KRAS-Mutant CRC Cells, Which Inhibits Proliferation. [Adapted from “KRAS Signaling Pathways”, from BioRender.com (2022)]. Abbreviations: δT3, δ-Tocotrienol; CRC, colorectal cancer

The *KRAS* gene codes for the K-RAS protein, a small guanosine triphosphate (GTP)ase.^83^ In healthy cells, hydrolysis of GTP at the G-domain of RAS isoforms can mediate downstream cell signal transduction pathways, namely the PI3K-Akt and RAS-RAF-MEK pathways.^84^ The binding of an extracellular ligand to the transmembrane epidermal growth factor receptor (EGFR) activates the tyrosine kinase (TK) receptor, which in turn stimulates guanine nucleotide exchange factors (GEFs). Activation of GEFs conveys a signal to the K-RAS protein, which activates the RAS-RAF-MEK pathway. Finally, the active form of RAS-GTP is hydrolyzed into the inactive form by the auxiliary proteins (GTPase-activating proteins (GAPs).^85^ However, mutated K-RAS protein promotes cancer growth, proliferation, and differentiation.^86^ So, in cancer cells with mutated *KRAS* genes, the GTPase activity and GAPs’ responsiveness to K-RAS protein become impaired, allowing a swift exchange of guanosine diphosphate (GDP) for GTP and allowing K-RAS to remain active in cancer cells. Furthermore, *KRAS* mutation also impacts the PI3K/Akt/mTOR cascade by inhibiting PI3K activity, allowing its substrate, phosphatidylinositol-3,4,5-triphosphate (PIP-3), to accumulate, resulting in the hyperphosphorylation of Akt protein.^87^ The pharmacological management of *KRAS*-mutant tumors is not easy due to the diminutive size and limited binding sites on the K-RAS protein.^88^ Nonetheless, γT3 has been identified as a potent inhibitor of CRC cell proliferation, irrespective of whether the *KRAS* gene is wild-type or mutated. Notably, treatment involving γT3 markedly suppressed cell proliferation **(**Figure 2**)**, with a higher sensitivity being observed in cell lines carrying mutated *KRAS* than in those with wild-type *KRAS*.^54^ These findings strongly imply that targeting the K-RAS protein could present a promising avenue for effective cancer therapy.

## INDUCTION OF CELL DEATH THROUGH APOPTOSIS AND PARAPTOSIS

Several cell death pathways can be triggered in normal or cancer cells, such as apoptosis, paraptosis, and necrosis. Although all 3 forms of cell death are programmed cell death pathways, only apoptosis is a caspase-dependent cell death pathway.^89–91^ Necrosis and proptosis are caspase-independent cell death pathways.^92^^,^^93^ The apoptosis-like characteristics (such as nuclear fragmentation, apoptotic body formation, and chromatin condensation) are absent in cells undergoing paraptosis.^94^ In cells undergoing paraptosis, huge cytoplasmic vacuolation can be observed, including enlargement of the endoplasmic reticulum and mitochondria without autophagic vacuoles.^95^

Several mechanisms have been suggested for how γT3 and δT3 activate the apoptosis pathway in cancer cells, such as colon cancer cells. For instance, exposing HT29 human colon cancer cells to γT3 reduced the levels of NF-κB/p65, which impeded the nuclear translocation of NF-κB/p65 and triggered apoptosis and cell cycle arrest in these cells.^69^ This was not surprising, as decreased levels of NF-κB/p65 have been linked to the induction of apoptosis and cell cycle arrest in cancer cells.^54^ Hence, the anticancer effects of γT3 may stem from its regulation of the NF-κB pathway. In a separate study, γT3 reduced the viability of HT29 human colon cancer cells by inhibiting the β-catenin/Tcf signaling pathway, which reduced the expression of *c-myc*, *cyclin D1*, and *survivin* genes.^61^ Some researchers have postulated that γT3 might activate apoptosis through the Bcl2 pathway, as modifications to Bcl2 proteins have been associated with activating both initiator caspase 9 and effector caspase 3.^96^

Gamma-T3 also induced paraptosis-like cell death in CRC cells by inhibiting the expression of *β-catenin*, *cyclin D1*, and *c-jun*,^60^ which suggests that the γT3-induced apoptosis-like cell death in these CRC cells may be linked to inhibition of the Wnt signaling pathway. A similar effect was observed in SW620 human CRC cells treated with δT3, which was associated with vacuolation caused by swelling and fusion of mitochondria and/or the endoplasmic reticulum (ER), as well as reduction of β-catenin, Wnt-1, cyclin D1, c-jun, and MMP-7 protein, which were not associated with activation of caspase 3.^65^ These findings imply that the anti-proliferative and pro-apoptotic effects of γT3 exposure can engage several programmed cell death pathways.

## CELL CYCLE ARREST

**Figure 3.**
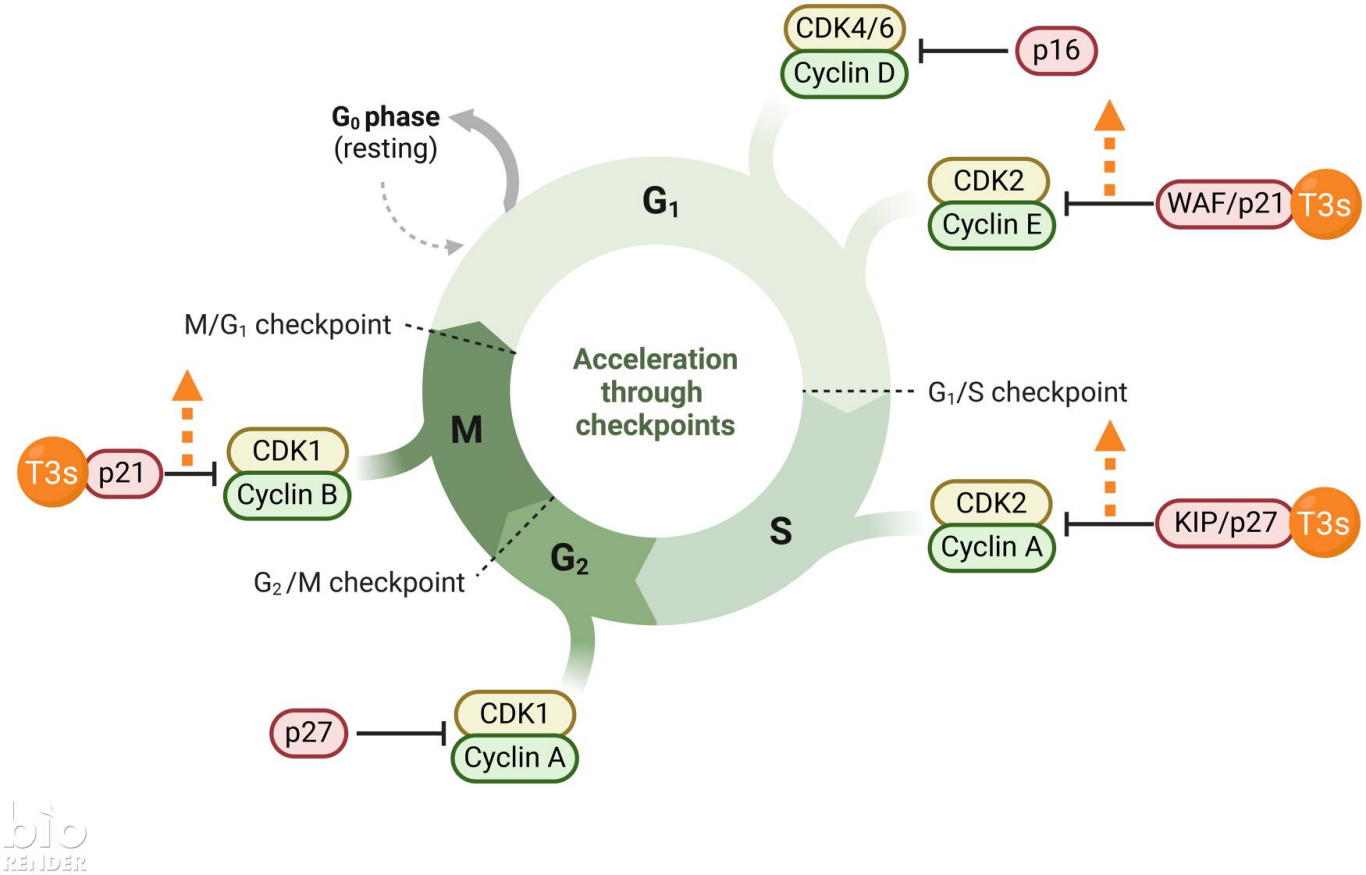
The Effects of Tocotrienol Cell Cycle Regulation That Affect Cell Proliferation. (Adapted from “Cell Cycle Deregulation in Cancer”, from BioRender.com [2022]). Abbreviation: δT3, δ-Tocotrienol

Cell cycle arrest is a stage within the cell cycle in which the cell ceases active replication.^97^ Usually, cells employ this state to facilitate DNA repair before resuming growth.^98^^,^^99^ However, in cases of severe DNA damage, cells might engage alternative signaling pathways to prompt either cell senescence or apoptosis, thereby preventing uncontrolled growth.^100^^,^^101^ A combination of γT3, atorvastatin (ATST), and celecoxib (CXIB) was reported to inhibit the proliferation of HT29 and HCT116 human CRC cells by arresting the cell cycle at the G0/G1 phase.^67^ Exposure to just ATST increased the levels of HMG-CoA reductase and reduced the levels of membrane-bound RhoA in these CRC cells, but the presence of γT3 negated both of these effects.^67^ A recent study revealed that combining γT3 and 6-gingerol (6G) induced differential expression of several genes involved in cell cycle arrests, such as *ATF6, DDIT3, GADD34, FOXM1, CDK1,* and *p21* in SW837 CRC cells (Figure 3).^48^ The synergistic anticancer effects of γT3 and 6G were reported to be augmented by several molecular events, including suppression of the Wnt signaling pathway, initiation of the endoplasmic reticulum–unfolded protein response, mitochondrial dysfunction, disruption of DNA repair mechanisms, and deactivation of the cell cycle progression through the downregulation of pivotal proliferation-related genes such as *FOXM1* and its subsequent downstream genes.^48^^,^^102^

## TELOMERASE INHIBITION

**Figure 4.**
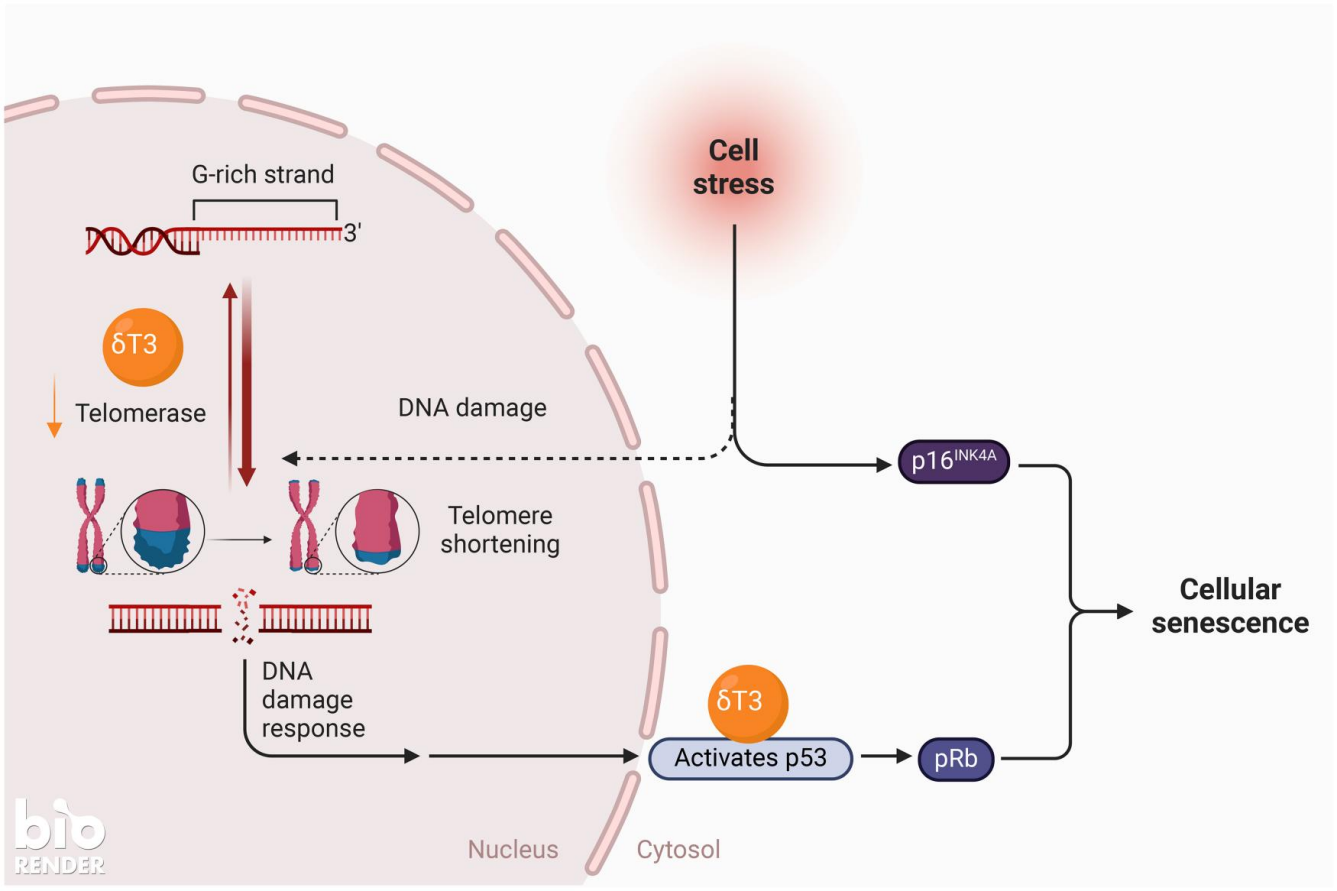
A Proposed Mechanism for How δ-Tocotrienol May Reduce Telomerase Activity, Which Mediates the Stress Response in Cancer. (Adapted from “Telomerase-Mediated Stress Response in Cancer”, from BioRender.com [2022]). Abbreviation: δT3, δ-Tocotrienol

Telomeres are highly preserved heterochromatic structures with long stretches containing repetitive DNA sequences at the extremities of chromosomes, which protect chromosomes and ensure proper chromosome replication.^103–105^ Telomeric DNA is reported to shorten upon each cell replication, which continues until the telomeres reach a critical length, triggering cell cycle arrest, senescence or apoptotic cell death.^106^ Telomere shortening is often linked to cellular ageing,^107^^,^^108^ but the effect of telomere shortening and telomeric repeats on tissue aging is unknown. However, some evidence suggests that telomere shortening may act as a molecular clock that triggers senescence.^109^^,^^110^ Critical telomere attrition may eventually trigger the DNA damage response (DDR), which induces cell cycle arrest and replicative senescence or apoptosis via the p53 or Rb tumor suppressor pathways (Figure 4).^106^^,^^109^ In general, telomerase is usually undetectable in most normal tissues,^110^ which presents a potential avenue for cancer therapy. Since most cancer cells exhibit substantial telomerase activity, inhibiting telomerase using natural bioactive agents or dietary components could offer an innovative approach to preventing cancer without causing harm to normal cells. Studies have shown that bioactive compounds, including T3s, can impede telomerase activity within cancer cells, consequently restraining their proliferation.^111^^,^^112^ However, there is a need to do more research to provide more substantial evidence to support this claim.

## SYNERGISTIC ACTIONS WITH ANTICANCER AGENTS

The concept of synergistic interactions between drugs and natural bioactive compounds has been a concern in biomedicine. Understanding the interactions between these elements is becoming increasingly crucial for assuring successful treatment regimens in complicated diseases such as cancers.^113^ The combination of low-dose jerantinine B (JB, a novel indole alkaloid) and δT3 caused dose-dependent growth inhibition in HT29 human CRC cells via multiple mechanisms, including double-stranded DNA damage and disruption of the microtubule network, and treatment increased the expression of caspase 8, 9 and 3 activities.^51^ A combination of JB and δT3 induced similar anticancer effects in human glioblastoma cells.^114^ Anticancer effects were also observed in combinations of alkaloid extracts from *Ficus fistulosa* with γT3 or δT3, which inhibited the proliferation of HT29 cells.^52^ These findings suggest that combining natural bioactive compounds such as γT3 or δT3 with anticancer drugs may target specific proteomes that result in pleiotropic effects, through the activation of multiple pathways and doubling of the inhibition of cancer cell growth. However, further research is needed to establish a value-added cancer chemotherapeutic regimen.

## SUPPRESSION OF METASTASIS AND ANGIOGENESIS

Metastasis is a complex phenomenon that leads to exceedingly aggressive and progressed cancer to its final stage,^115^^,^^116^ through several processes, including epithelial‐to‐mesenchymal transition (EMT) and the mesenchymal‐to‐epithelial transition (MET).^117^^,^^118^ Cancer stem cells (CSCs) are principal participants in metastasis, and it has been proposed that these cells possess epithelial–mesenchymal plasticity.^119^ The microenvironment at the primary and secondary tumor sites is critical in determining whether a cancer cell can successfully leave its original site and establish itself at a new location.^120^^,^^121^ However, tumor growth and spread require a process known as angiogenesis, which is the process through which new blood vessels are developed from pre-existing vessels.^122^^,^^123^ Angiogenesis is an intricate, multifaceted process essential for the continued growth of the primary tumor, facilitating metastasis and supporting subsequent metastatic tumor growth and progression. Pro- and anti-angiogenic factors regulate this process. During tumor development, angiogenesis is prompted when an imbalance between the pro-and anti-angiogenic factors confers an “angiogenic switch”.^124^

Several approaches to inhibit metastasis and angiogenesis have been proposed and tested, including modulation of the tumor microenvironment.^125^ Several cytokines and growth factors are required for angiogenesis to take place. Gamma-T3 and δT3 are reported to inhibit the production of many of the cytokines and growth factors involved in angiogenesis, such as transforming growth factor-beta (TGF-β), tumor necrosis factor-alpha (TNF-α), interleukin (IL)-6, IL-10, IL-35, IL-1β, and vascular endothelial growth factor (VEGF).^126^^,^^127^ Tocotrienols were reported to inhibit angiogenesis in the tumor microenvironment via regulation of hypoxia-inducible factor-1α (HIF-1α) and its downstream target, the VEGF, and other angiogenic factors such as IL-8 and cyclooxygenase 2 (COX-2) in DLD-1 human CRC cells.^128^ The anti-angiogenic effect of δT3 may be due to the modulation of growth factor–dependent phosphatidylinositol-3 kinase (PI3K)/phosphoinositide-dependent protein kinase (PDK)/Akt signaling, as well as the induction of a stress response in endothelial cells.^127^^,^^128^ In addition, γT3 and δT3 were found to be more potent anticancer agents than α-Toc in the DLD-1 CRC cells and the human umbilical vein endothelial cells (HUVECs) in terms of anti-proliferation, anti-metastasis and vasculogenesis, and anti-angiogenesis.^68^

## MODIFICATION OF IMMUNITY

A weak immune system can exacerbate the progression of malignancies.^129–131^ It has been proposed that some of the cytokines produced by tumors and their surrounding tissues may play a significant role in dictating the immune escape mechanisms adopted by tumors.^132^^,^^133^ NF-κB is a protein with a vital function in inflammation and cancer development,^134–136^ and inhibition of NF-κB has been shown to reduce cancer progression and inflammation.^137^ Tocotrienols also possess anti-inflammatory^138–140^ and immune-enhancing activities.^141^^,^^142^ For example, daily supplementation of δT3 reduced TNF-α–induced activation of NF-κB in mice with colitis-induced colon cancer through decreased phosphorylation and degradation of the NF-κB polypeptide gene enhancer in B-cells inhibitor, alpha (IκBα) in the cytosol, and inhibited phosphorylation of p65 in the nucleus.^72^ Furthermore, δT3 inhibited TNFα-induced phosphorylation of the transforming growth factor β-activated kinase 1 (TAK1), a crucial upstream kinase essential for activating NF-κB in these mice.^72^ In contrast, δT3 enhanced the expression of A20 and CYLD, which are negative regulators of NF-κB. These findings suggest that δT3 can inhibit NF-κB activity and suppress cancer progression.

## FURTHER INSIGHTS INTO THE PROPOSED ANTI-COLORECTAL CANCER MECHANISMS OF GAMMA AND DELTA TOCOTRIENOLS

### Modification of tumor–stromal interactions

The relationship between tumors and stromal cells involves various soluble substances in the tumor microenvironment and cell–cell interactions.^143^ There is growing evidence to suggest that tumor–stromal interactions can increase or decrease the susceptibility of tumors to treatment.^144^ Stromal cell–like macrophages and fibroblasts may play a key role in CRC, as do angiogenic factors such as TNF-α, matrix metalloproteinase-2 (MMP-2), and VEGF-A, as these can increase macrophage recruitment, extracellular matrix remodeling, and endothelial cell proliferation.^145^ A recent study found that a δT3-rich diet reduced the effect of stromal cells on colon cancer development in a mouse model.^50^

### Regulation of noncoding RNAs

Micro-RNAs (miRNAs) and long noncoding RNAs (lncRNAs) are noncoding RNAs that are 21–23 and more than 200 nucleotides long, respectively, that may be targeted for anticancer drug development.^146^^,^^147^ Although the importance and mechanisms of action of miRNAs and lncRNAs are well documented, their possible role in cancer development is only starting to be recognized.^148^^,^^149^ For instance, human breast cancer (BC) cells treated with δT3 had considerably lower levels of miRNA-429 expression than untreated cells.^150^ Furthermore, δT3-treated human BC cells transfected with control miRNAs also showed significantly higher caspase-3 activity and apoptosis compared with cells transfected with an inhibitor of the miRNA-429, and the X-linked inhibitor of apoptosis protein (XIAP) was discovered to be a target gene for miRNA-429.^150^ Exposure to δT3 induced a dose- and time-dependent increase in the expression of miR-34a in lung cancer cell lines (H1650 and A549), but a combination of δT3 with an inhibitor of miRNA-34 markedly reduced cell proliferation.^151^ It is possible that γT3 and δT3 may act by modulating noncoding RNAs such as miRNAs and lncRNAs. Currently, very limited studies have reported on the role of T3s in regulating the expression of noncoding RNAs in cancer cells.

### Suppression of cancer stem cells

The idea that cancer stem cells (CSCs) play a vital role in the initiation, development, and metastasis of malignancies is gaining momentum. As a result, CSCs are being explored as a potential target for future cancer therapeutics. The glycoprotein CD133 has been identified as a CSC marker for CRC.^152^^,^^153^ The CD133^+^ cells comprised 2.5% of tumor cells that appear to have lost their ability to differentiate.^154^ This is a growing area of research interest, but to date, the effect of T3s in regulating the expression of CD133 in colon cancer cells has not been reported. However, γT3 has been reported to inhibit the expression of cancer stem cell markers in prostate cancer.^155^

### Modification of microflora

Gut microbiota can play an essential role in the progression of CRC.^156^ The cancer-preventive properties of T3s and its metabolite δT3-13′-carboxychromanol (δT3-13′) are well recognized,^72^ but their impact on the gut flora during carcinogenesis is still not fully elucidated. Both δT3 and δT3-13′ were reported to improve the levels of 2 potentially helpful gut bacteria, ie, *Lactococcus* and *Bacteroides*, and reduce carcinogenesis and decrease pro-inflammatory cytokines in a mouse model of colon cancer.^71^ These findings suggest that δT3 and δT3-13′ may play a role in T3s’ anticancer and gut microbial regulation.

## CONCLUSION

The evidence in the literature show that T3s, especially γT3 and δT3, exhibit potent anticancer properties. The growing popularity of research into γT3 and δT3 will almost certainly help enhance the understanding of the molecular mechanisms underlying their anticancer activities. In addition, the promising anticancer effects observed in several recent preclinical investigations may provide impetus for the development of clinical trials to explore the anticancer potential of γT3 and δT3.

## Supplementary Material

nuae108_Supplementary_Data

## References

[1] Sung H , Ferlay J, Siegel RL, et al Global cancer statistics 2020: GLOBOCAN estimates of incidence and mortality worldwide for 36 cancers in 185 countries. CA Cancer J Clin. 2021;71(3):209–249. 10.3322/caac.21660 33538338

[2] Rawla P , Sunkara T, Barsouk A. Epidemiology of colorectal cancer: incidence, mortality, survival, and risk factors. Prz Gastroenterol. 2019;14(2):89–103. 10.5114/pg.2018.81072 31616522 PMC6791134

[3] Mármol I , Sánchez-de-Diego C, Pradilla Dieste A, Cerrada E, Rodriguez Yoldi MJ. Colorectal carcinoma: a general overview and future perspectives in colorectal cancer. Int J Mol Sci. 2017;18(1):197. 10.3390/ijms18010197 28106826 PMC5297828

[4] Xi Y , Xu P. Global colorectal cancer burden in 2020 and projections to 2040. Transl Oncol. 2021;14(10):101174. 10.1016/j.tranon.2021.101174 34243011 PMC8273208

[5] Thrumurthy SG , Thrumurthy SSD, Gilbert CE, Ross P, Haji A. Colorectal adenocarcinoma: risks, prevention and diagnosis. BMJ. 2016;354:I3590. 10.1136/bmj.i3590 27418368

[6] Konishi T , Watanabe T, Kishimoto J, Kotake K, Muto T, Nagawa H; Japanese Society for Cancer of the Colon and Rectum. Prognosis and risk factors of metastasis in colorectal carcinoids: results of a nationwide registry over 15 years. Gut. 2007;56(6):863–868. 10.1136/gut.2006.109157 17213340 PMC1954860

[7] Corless CL , Fletcher JA, Heinrich MC. Biology of gastrointestinal stromal tumors. J Clin Oncol. 2004;22(18):3813–3825. 10.1200/JCO.2004.05.140 15365079

[8] Times M. Colorectal lymphoma. Clin Colon Rectal Surg. 2011;24(3):135–141. 10.1055/s-0031-1285997 22942795 PMC3311500

[9] Strate LL , Syngal S. Hereditary colorectal cancer syndromes. Cancer Causes Control. 2005;16(3):201–213. 10.1007/s10552-004-3488-4 15947872

[10] Johnson CM , Wei C, Ensor JE, et al Meta-analyses of colorectal cancer risk factors. Cancer Causes Control. 2013;24(6):1207–1222. 10.1007/s10552-013-0201-5 23563998 PMC4161278

[11] Inamura K , Hamada T, Bullman S, Ugai T, Yachida S, Ogino S. Cancer as microenvironmental, systemic and environmental diseases: opportunity for transdisciplinary microbiomics science. Gut. 2022;71(10):2107–2122. 10.1136/gutjnl-2022-327209 PMC9834441 35820782

[12] Ogino S , Nowak JA, Hamada T, Milner DA, Nishihara R. Insights into pathogenic interactions among environment, host, and tumor at the crossroads of molecular pathology and epidemiology. Annu Rev Pathol. 2019;14(1):83–103. 10.1146/annurev-pathmechdis-012418-012818 30125150 PMC6345592

[13] Pacheco-Pérez LA , Ruíz-González KJ, de-la-Torre-Gómez ACG, Guevara-Valtier MC, Rodríguez-Puente LA, Gutiérrez-Valverde JM. Environmental factors and awareness of colorectal cancer in people at familial risk. Rev Lat Am Enfermagem. 2019;27:e3195. 31618388 10.1590/1518-8345.3082.3195 PMC6792335

[14] Rattray NJW , Charkoftaki G, Rattray Z, Hansen JE, Vasiliou V, Johnson CH. Environmental influences in the etiology of colorectal cancer: the premise of metabolomics. Curr Pharmacol Rep. 2017;3(3):114–125. 10.1007/s40495-017-0088-z 28642837 PMC5475285

[15] Bishehsari F , Mahdavinia M, Vacca M, Malekzadeh R, Mariani-Costantini R. Epidemiological transition of colorectal cancer in developing countries: environmental factors, molecular pathways, and opportunities for prevention. World J Gastroenterol. 2014;20(20):6055–6072. 10.3748/wjg.v20.i20.6055 24876728 PMC4033445

[16] Inamura K. Colorectal cancers: an update on their molecular pathology. Cancers (Basel). 2018;10(1):26. 10.3390/cancers10010026 29361689 PMC5789376

[17] Jin D , Lu Y, Wu W, et al Diet-wide association, genetic susceptibility and colorectal cancer risk: a prospective cohort study. Nutrients. 2023;15(22):4801. 10.3390/nu15224801 38004195 PMC10674290

[18] Sepulveda AR , Hamilton SR, Allegra CJ, et al Molecular biomarkers for the evaluation of colorectal cancer: guideline from the American Society for Clinical Pathology, College of American Pathologists, Association for Molecular Pathology, and the American Society of Clinical Oncology. J Clin Oncol. 2017;35(13):1453–1486. 10.1200/JCO.2016.71.9807 28165299

[19] Ye DJ , Zhang JM. Research development of the relationship between thymidine phosphorylase expression and colorectal carcinoma. Cancer Biol Med. 2013;10(1):10–15. 10.7497/j.issn.2095-3941.2013.01.002 23691439 PMC3643685

[20] Tampakis A , Tampaki EC, Nonni A, et al L1CAM expression in colorectal cancer identifies a high-risk group of patients with dismal prognosis already in early-stage disease. Acta Oncol. 2020;59(1):55–59. 10.1080/0284186X.2019.1667022 31532272

[21] Hammond WA , Swaika A, Mody K. Pharmacologic resistance in colorectal cancer: a review. Ther Adv Med Oncol. 2016;8(1):57–84. 10.1177/1758834015614530 26753006 PMC4699262

[22] Eisenmann ED , Talebi Z, Sparreboom A, Baker SD. Boosting the oral bioavailability of anticancer drugs through intentional drug–drug interactions. Basic Clin Pharmacol Toxicol. 2022;130(suppl 1):23–35. 10.1111/bcpt.13623 34117715 PMC8665934

[23] Stuurman FE , Nuijen B, Beijnen JH, Schellens JHM. Oral anticancer drugs: mechanisms of low bioavailability and strategies for improvement. Clin Pharmacokinet. 2013;52(6):399–414. 10.1007/s40262-013-0040-2 23420518

[24] Nurgali K , Jagoe RT, Abalo R. Editorial: adverse effects of cancer chemotherapy: anything new to improve tolerance and reduce sequelae? Front Pharmacol. 2018;9:245. 10.3389/fphar.2018.00245 29623040 PMC5874321

[25] Qureshi AA , Zuvanich EG, Khan DA, Mushtaq S, Silswal N, Qureshi N. Proteasome inhibitors modulate anticancer and anti-proliferative properties via NF-κB signaling, and ubiquitin–proteasome pathways in cancer cell lines of different organs. Lipids Health Dis. 2018;17(1):62. 10.1186/s12944-018-0697-5 29606130 PMC5879737

[26] Ahmed K , Zaidi SF, Cui ZG, Zhou D, Saeed SA, Inadera H. Potential proapoptotic phytochemical agents for the treatment and prevention of colorectal cancer. Oncol Lett. 2019;18(1):487–498. 10.3892/ol.2019.10349 31289520 PMC6540497

[27] Ahsan H , Ahad A, Siddiqui WA. A review of characterization of tocotrienols from plant oils and foods. J Chem Biol. 2015;8(2):45–59. 10.1007/s12154-014-0127-8 25870713 PMC4392014

[28] Galli F , Azzi A, Birringer M, et al Vitamin E: emerging aspects and new directions. Free Radic Biol Med. 2017;102:16–36. 10.1016/j.freeradbiomed.2016.09.017 27816611

[29] Kamal-Eldin A , Appelqvist LÅ. The chemistry and antioxidant properties of tocopherols and tocotrienols. Lipids. 1996;31(7):671–701. 10.1007/BF02522884 8827691

[30] Szewczyk K , Chojnacka A, Górnicka M. Tocopherols and tocotrienols—bioactive dietary compounds; what is certain, what is doubt? Int J Mol Sci. 2021;22(12):6222. 10.3390/ijms22126222 34207571 PMC8227182

[31] Prasad K. Tocotrienols and cardiovascular health. Curr Pharm Des. 2011;17(21):2147–2154. 10.2174/138161211796957418 21774782

[32] Selvaraju TR , Khaza’ai H, Vidyadaran S, Abd Mutalib MS, Vasudevan R. The neuroprotective effects of tocotrienol rich fraction and alpha tocopherol against glutamate injury in astrocytes. Bosn J Basic Med Sci. 2014;14(4):195–204. 25428670 10.17305/bjbms.2014.4.91 PMC4333972

[33] Zafar H , Mirza IA, Hussain W, Fayyaz M. Comparative efficacy of tocotrienol and tocopherol for their anti diabetic effects. Biomed J Sci Tech Res. 2021;38(5):30835–30840. 10.26717/BJSTR.2021.38.006226

[34] Pang KL , Chin KY. The role of tocotrienol in protecting against metabolic diseases. Molecules. 2019;24(5):923. 10.3390/molecules24050923 30845769 PMC6429133

[35] Serbinova E , Kagan V, Han D, Packer L. Free radical recycling and intramembrane mobility in the antioxidant properties of alpha-tocopherol and alpha-tocotrienol. Free Radic Biol Med. 1991;10(5):263–275. 10.1016/0891-5849(91)90033-y 1649783

[36] Garg S , Garg TK, Miousse IR, et al Effects of gamma-tocotrienol on partial-body irradiation-induced intestinal injury in a nonhuman primate model. Antioxidants (Basel). 2022;11(10):1895. 10.3390/antiox11101895 36290618 PMC9598988

[37] Aggarwal V , Kashyap D, Sak K, et al Molecular mechanisms of action of tocotrienols in cancer: recent trends and advancements. Int J Mol Sci. 2019;20(3):656. 10.3390/ijms20030656 30717416 PMC6386883

[38] Montagnani Marelli M , Marzagalli M, Fontana F, Raimondi M, Moretti RM, Limonta P. Anticancer properties of tocotrienols: a review of cellular mechanisms and molecular targets. J Cell Physiol. 2019;234(2):1147–1164. 10.1002/jcp.27075 30066964

[39] Ling MT , Luk SU, Al-Ejeh F, Khanna KK. Tocotrienol as a potential anticancer agent. Carcinogenesis. 2012;33(2):233–239. 10.1093/carcin/bgr261 22095072

[40] Kannappan R , Gupta SC, Kim JH, Aggarwal BB. Tocotrienols fight cancer by targeting multiple cell signaling pathways. Genes Nutr. 2012;7(1):43–52. 10.1007/s12263-011-0220-3 21484157 PMC3250528

[41] Khalid AQ , Bhuvanendran S, Magalingam KB, Ramdas P, Kumari M, Radhakrishnan AK. Clinically relevant genes and proteins modulated by tocotrienols in human colon cancer cell lines: systematic scoping review. Nutrients. 2021;13(11):4056. 10.3390/nu13114056 34836311 PMC8625890

[42] Gao X , Wilde PE, Lichtenstein AH, Bermudez OI, Tucker KL. The maximal amount of dietary alpha-tocopherol intake in U.S. adults (NHANES 2001–2002). J Nutr. 2006;136(4):1021–1026. 10.1093/jn/136.4.1021 16549468

[43] Ahsan H , Ahad A, Iqbal J, Siddiqui WA. Pharmacological potential of tocotrienols: a review. Nutr Metab (Lond). 2014;11(1):52. 10.1186/1743-7075-11-52 25435896 PMC4247006

[44] Wu K , Willett WC, Chan JM, et al A prospective study on supplemental vitamin E intake and risk of colon cancer in women and men. Cancer Epidemiol Biomarkers Prev. 2002;11(11):1298–1304. 10.1016/s0739-5930(08)79000-7 12433706

[45] Hiura Y , Tachibana H, Arakawa R, et al Specific accumulation of γ- and δ-tocotrienols in tumor and their antitumor effect in vivo. J Nutr Biochem. 2009;20(8):607–613. 10.1016/j.jnutbio.2008.06.004 18824342

[46] Szulczewska-Remi A , Nogala-Kalucka M. Studies on the growth inhibiting and non-cytotoxic effects of tocotrienols on selected cancer cell lines. Acta Sci Pol Technol Aliment. 2020;19(2):139–147. 10.17306/J.AFS.0787 32600010

[47] Husain K , Zhang A, Shivers S, et al Chemoprevention of azoxymethane-induced colon carcinogenesis by delta-tocotrienol. Cancer Prev Res (Phila). 2019;12(6):357–366. 10.1158/1940-6207.CAPR-18-0290 30940630

[48] Yusof KM , Makpol S, Fen LS, Jamal R, Wan Ngah WZ. Suppression of colorectal cancer cell growth by combined treatment of 6-gingerol and γ-tocotrienol via alteration of multiple signalling pathways. J Nat Med. 2019;73(4):745–760. 10.1007/s11418-019-01323-6 31177355

[49] Balagoni H , Shipley LC, Lightner J, Stone W, Palau V, Krishnan K. Cytotoxic effects of metformin and gamma-tocotrienol on colon cancer cells: 153. Am J Gastroenterol. 2017;112:S77.

[50] Wada S , Naito Y, Matsushita Y, et al δ-Tocotrienol suppresses tumorigenesis by inducing apoptosis and blocking the COX-2/PGE2 pathway that stimulates tumor–stromal interactions in colon cancer. J Funct Foods. 2017;35:428–435. 10.1016/j.jff.2017.06.002

[51] Abubakar IB , Lim KH, Kam TS, Loh HS. Synergistic cytotoxic effects of combined δ-tocotrienol and jerantinine B on human brain and colon cancers. J Ethnopharmacol. 2016;184:107–118. 10.1016/j.jep.2016.03.004 26947901

[52] Eitsuka T , Tatewaki N, Nishida H, Nakagawa K, Miyazawa T. A combination of δ-tocotrienol and ferulic acid synergistically inhibits telomerase activity in DLD-1 human colorectal adenocarcinoma cells. J Nutr Sci Vitaminol (Tokyo). 2016;62(5):281–287. 10.3177/jnsv.62.281 27928113

[53] Jang Y , Park NY, Rostgaard-Hansen AL, Huang J, Jiang Q. Vitamin E metabolite 13'-carboxychromanols inhibit pro-inflammatory enzymes, induce apoptosis and autophagy in human cancer cells by modulating sphingolipids and suppress colon tumor development in mice. Free Radic Biol Med. 2016;95:190–199. 10.1016/j.freeradbiomed.2016.03.018 27016075 PMC4867259

[54] Prasad S , Gupta SC, Tyagi AK, Aggarwal BB. γ-Tocotrienol suppresses growth and sensitises human colorectal tumours to capecitabine in a nude mouse xenograft model by down-regulating multiple molecules. Br J Cancer. 2016;115(7):814–824. 10.1038/bjc.2016.257 27575851 PMC5046209

[55] Abubakar IB , Lim KH, Loh HS. Alkaloid extracts of *Ficus* species and palm oil-derived tocotrienols synergistically inhibit proliferation of human cancer cells. Nat Prod Res. 2015;29(22):2137–2140. 10.1080/14786419.2014.991927 25515603

[56] Gu W , Prasadam I, Yu M, et al Gamma tocotrienol targets tyrosine phosphatase SHP2 in mammospheres resulting in cell death through RAS/ERK pathway. BMC Cancer. 2015;15(1):609. 10.1186/s12885-015-1614-1 26315028 PMC4552156

[57] Shibata A , Nakagawa K, Tsuduki T, Miyazawa T. δ-Tocotrienol treatment is more effective against hypoxic tumor cells than normoxic cells: potential implications for cancer therapy. J Nutr Biochem. 2015;26(8):832–840. 10.1016/j.jnutbio.2015.02.011 25979648

[58] Yusof KM , Makpol S, Jamal R, Harun R, Mokhtar N, Ngah WZW. γ-tocotrienol and 6-gingerol in combination synergistically induce cytotoxicity and apoptosis in HT-29 and SW837 human colorectal cancer cells. Molecules. 2015;20(6):10280–10297. 10.3390/molecules200610280 26046324 PMC6272690

[59] Forster GM , Raina K, Kumar A, et al Rice varietal differences in bioactive bran components for inhibition of colorectal cancer cell growth. Food Chem. 2013;141(2):1545–1552. 10.1016/j.foodchem.2013.04.020 23790950 PMC3720235

[60] Zhang JS , Li DM, Ma Y, et al γ-Tocotrienol induces paraptosis-like cell death in human colon carcinoma SW620 cells. PLoS One. 2013;8(2):e57779. 10.1371/journal.pone.0057779 23469066 PMC3585143

[61] Xu W , Du M, Zhao Y, Wang Q, Sun W, Chen B. γ-Tocotrienol inhibits cell viability through suppression of β-catenin/Tcf signaling in human colon carcinoma HT-29 cells. J Nutr Biochem. 2012;23(7):800–807. 10.1016/j.jnutbio.2011.04.003 21852086

[62] Yang Z , Lee MJ, Zhao Y, Yang CS. Metabolism of tocotrienols in animals and synergistic inhibitory actions of tocotrienols with atorvastatin in cancer cells. Genes Nutr. 2012;7(1):11–18. 10.1007/s12263-011-0233-y 21590436 PMC3250521

[63] Yue MA , He N, Zhang J, Li D, Liu Y, Zhang J. Experimental study on delta-tocotrienol inhibits the Wnt pathway in the colon cancer cell SW620. Wei Sheng Yan Jiu. 2012;41(6):900–904. 23424864

[64] de Mesquita ML , Araújo RM, Bezerra DP, et al Cytotoxicity of δ-tocotrienols from *Kielmeyera coriacea* against cancer cell lines. Bioorg Med Chem. 2011;19(1):623–630. 10.1016/j.bmc.2010.10.044 21094611

[65] Zhang JS , Li DM, He N, et al A paraptosis-like cell death induced by δ-tocotrienol in human colon carcinoma SW620 cells is associated with the suppression of the Wnt signaling pathway. Toxicology. 2011;285(1–2):8–17. 10.1016/j.tox.2011.03.011 21453743

[66] Shibata A , Nakagawa K, Sookwong P, Tsuduki T, Asai A, Miyazawa T. alpha-Tocopherol attenuates the cytotoxic effect of delta-tocotrienol in human colorectal adenocarcinoma cells. Biochem Biophys Res Commun. 2010;397(2):214–219. 10.1016/j.bbrc.2010.05.087 20493172

[67] Yang Z , Xiao H, Jin H, Koo PT, Tsang DJ, Yang CS. Synergistic actions of atorvastatin with γ-tocotrienol and celecoxib against human colon cancer HT29 and HCT116 cells. Int J Cancer. 2010;126(4):852–863. 10.1002/ijc.24766 19626588 PMC2981082

[68] Shibata A , Nakagawa K, Sookwong P, Tsuduki T, Oikawa S, Miyazawa T. δ-Tocotrienol suppresses VEGF induced angiogenesis whereas α-tocopherol does not. J Agric Food Chem. 2009;57(18):8696–8704. 10.1021/jf9012899 19702331

[69] Xu WL , Liu JR, Liu HK, et al Inhibition of proliferation and induction of apoptosis by gamma-tocotrienol in human colon carcinoma HT-29 cells. Nutrition. 2009;25(5):555–566. 10.1016/j.nut.2008.10.019 19121919

[70] Eitsuka T , Nakagawa K, Miyazawa T. Down-regulation of telomerase activity in DLD-1 human colorectal adenocarcinoma cells by tocotrienol. Biochem Biophys Res Commun. 2006;348(1):170–175. 10.1016/j.bbrc.2006.07.029 16875674

[71] Yang C , Zhao Y, Im S, Nakatsu C, Jones-Hall Y, Jiang Q. Vitamin E delta-tocotrienol and metabolite 13'-carboxychromanol inhibit colitis-associated colon tumorigenesis and modulate gut microbiota in mice. J Nutr Biochem. 2021;89:108567. 10.1016/j.jnutbio.2020.108567 33347911

[72] Yang C , Jiang Q. Vitamin E δ-tocotrienol inhibits TNF-α-stimulated NF-κB activation by up-regulation of anti-inflammatory A20 via modulation of sphingolipid including elevation of intracellular dihydroceramides. J Nutr Biochem. 2019;64:101–109. 10.1016/j.jnutbio.2018.10.013 30471562 PMC6363855

[73] Qureshi AA , Reis JC, Papasian CJ, Morrison DC, Qureshi N. Tocotrienols inhibit lipopolysaccharide-induced pro-inflammatory cytokines in macrophages of female mice. Lipids Health Dis. 2010;9:143. 10.1186/1476-511X-9-143 21162750 PMC3016328

[74] Shibata A , Nakagawa K, Sookwong P, Tsuzuki T, Oikawa S, Miyazawa T. Tumor anti-angiogenic effect and mechanism of action of delta-tocotrienol. Biochem Pharmacol. 2008;76(3):330–339. 10.1016/j.bcp.2008.05.017 18599020

[75] Nakagawa K , Shibata A, Yamashita S, et al In vivo angiogenesis is suppressed by unsaturated vitamin E, tocotrienol1,2. J Nutr. 2007;137(8):1938–1943. 10.1093/jn/137.8.1938 17634267

[76] Vejle Hospital. Tocotrienol and Bevacizumab in Metastatic Colorectal Cancer. A Randomized Phase II Marker Trial. clinicaltrials.gov; 2022. Accessed May 8, 2023. https://clinicaltrials.gov/ct2/show/NCT04245865

[77] Raunkilde L , Hansen TF, Havelund BM, et al Delta tocotrienol as a supplement to FOLFOXIRI in first-line treatment of metastatic colorectal cancer. A randomized, double-blind, placebo-controlled phase II stud*y*. Acta Oncol. 2023;62(9):1066–1075. 10.21203/rs.3.rs-1110700/v1 37646150

[78] Duronio RJ , Xiong Y. Signaling pathways that control cell proliferation. Cold Spring Harb Perspect Biol. 2013;5(3):a008904. https://doi.org/10.1080/0284186X.2023.2249225 23457258 10.1101/cshperspect.a008904 PMC3578363

[79] Loganathan R , Radhakrishnan AK, Selvaduray KR, Nesaretnam K. Selective anti-cancer effects of palm phytonutrients on human breast cancer cells. RSC Adv. 2015;5(3):1745–1753. 10.1039/C4RA12343C

[80] Srivastava JK , Gupta S. Tocotrienol-rich fraction of palm oil induces cell cycle arrest and apoptosis selectively in human prostate cancer cells. Biochem Biophys Res Commun. 2006;346(2):447–453. 10.1016/j.bbrc.2006.05.147 16762318

[81] Tham SY , Loh HS, Mai CW, Fu JY. Tocotrienols modulate a life or death decision in cancers. Int J Mol Sci. 2019;20(2):372. 10.3390/ijms20020372 30654580 PMC6359475

[82] Liu HK , Wang Q, Li Y, et al Inhibitory effects of gamma-tocotrienol on invasion and metastasis of human gastric adenocarcinoma SGC-7901 cells. J Nutr Biochem. 2010;21(3):206–213. 10.1016/j.jnutbio.2008.11.004 19195866

[83] Jančík S , Drábek J, Radzioch D, Hajdúch M. Clinical relevance of KRAS in human cancers. J Biomed Biotechnol. 2010;2010(1):150960. 10.1155/2010/150960 20617134 PMC2896632

[84] Scheffzek K , Shivalingaiah G. Ras-specific GTPase-activating proteins—structures, mechanisms, and interactions. Cold Spring Harb Perspect Med. 2019;9(3):a031500. 10.1101/cshperspect 30104198 PMC6396337

[85] Ferreira A , Pereira F, Reis C, Oliveira MJ, Sousa MJ, Preto A. Crucial role of oncogenic *KRAS* mutations in apoptosis and autophagy regulation: therapeutic implications. Cells. 2022;11(14):2183. 10.3390/cells11142183 35883626 PMC9319879

[86] Takács T , Kudlik G, Kurilla A, Szeder B, Buday L, Vas V. The effects of mutant Ras proteins on the cell signalome. Cancer Metastasis Rev. 2020;39(4):1051–1065. 10.1007/s10555-020-09912-8 32648136 PMC7680337

[87] Sullivan KM , Kozuch PS. Impact of *KRAS* mutations on management of colorectal carcinoma. Patholog Res Int. 2011;2011:219309. 10.4061/2011/219309 21437184 PMC3062096

[88] Johnson CW , Reid D, Parker JA, et al The small GTPases K-Ras, N-Ras, and H-Ras have distinct biochemical properties determined by allosteric effects. J Biol Chem. 2017;292(31):12981–12993. 10.1074/jbc.M117.778886 28630043 PMC5546037

[89] Alberts B , Johnson A, Lewis J, Raff M, Roberts K, Walter P. Programmed cell death (apoptosis). In: Molecular Biology of the Cell. 4th ed. Garland Science; 2002. Accessed May 11, 2023. https://www.ncbi.nlm.nih.gov/books/NBK26873/

[90] Griffioen AW , Nowak-Sliwinska P. Programmed cell death lives. Apoptosis. 2022;27(9–10):619–621. 10.1007/s10495-022-01758-5 35943678 PMC9361233

[91] Wu SJ , Huang GY, Ng LT. γ-Tocotrienol induced cell cycle arrest and apoptosis via activating the Bax-mediated mitochondrial and AMPK signaling pathways in 3T3-L1 adipocytes. Food Chem Toxicol. 2013;59:501–513. 10.1016/j.fct.2013.06.011 23816832

[92] McCall K. Genetic control of necrosis—another type of programmed cell death. Curr Opin Cell Biol. 2010;22(6):882–888. 10.1016/j.ceb.2010.09.002 20889324 PMC2993806

[93] Fontana F , Raimondi M, Marzagalli M, Di Domizio A, Limonta P. The emerging role of paraptosis in tumor cell biology: perspectives for cancer prevention and therapy with natural compounds. Biochim Biophys Acta Rev Cancer. 2020;1873(2):188338. 10.1016/j.bbcan.2020.188338 31904399

[94] Sperandio S , Poksay KS, Schilling B, Crippen D, Gibson BW, Bredesen DE. Identification of new modulators and protein alterations in non-apoptotic programmed cell death. J Cell Biochem. 2010;111(6):1401–1412. 10.1002/jcb.22870 20830744 PMC5668132

[95] Hoa N , Myers MP, Douglass TG, et al Molecular mechanisms of paraptosis induction: implications for a non-genetically modified tumor vaccine. PLoS One. 2009;4(2):e4631. 10.1371/journal.pone.0004631 19247476 PMC2645013

[96] Sun W , Wang Q, Chen B, Liu J, Liu H, Xu W. Gamma-tocotrienol-induced apoptosis in human gastric cancer SGC-7901 cells is associated with a suppression in mitogen-activated protein kinase signalling. Br J Nutr. 2008;99(6):1247–1254. 10.1017/S0007114507879128 18081943

[97] Sadoughi F , Hallajzadeh J, Asemi Z, Mansournia MA, Alemi F, Yousefi B. Signaling pathways involved in cell cycle arrest during the DNA breaks. DNA Repair (Amst). 2021;98:103047. 10.1016/j.dnarep.2021.103047 33454524

[98] Kumari R , Jat P. Mechanisms of cellular senescence: cell cycle arrest and senescence associated secretory phenotype. Front Cell Dev Biol. 2021;9:645593. 10.3389/fcell.2021.645593 33855023 PMC8039141

[99] Campos A , Clemente-Blanco A. Cell cycle and DNA repair regulation in the damage response: protein phosphatases take over the reins. Int J Mol Sci. 2020;21(2):446. 10.3390/ijms21020446 31936707 PMC7014277

[100] Chao HX , Poovey CE, Privette AA, et al Orchestration of DNA damage checkpoint dynamics across the human cell cycle. Cell Syst. 2017;5(5):445–459.e5. 10.1016/j.cels.2017.09.015 29102360 PMC5700845

[101] Labi V , Erlacher M. How cell death shapes cancer. Cell Death Dis. 2015;6(3):e1675. 10.1038/cddis.2015.20 25741600 PMC4385913

[102] Yue M , Li S, Yan G, Li C, Kang Z. Paeoniflorin inhibits cell growth and induces cell cycle arrest through inhibition of FoxM1 in colorectal cancer cells. Cell Cycle. 2018;17(2):240–249. 10.1080/15384101.2017.1407892 29301438 PMC5884122

[103] Zhao Y , Sfeir AJ, Zou Y, et al Telomere extension occurs at most chromosome ends and is uncoupled from fill-in in human cancer cells. Cell. 2009;138(3):463–475. 10.1016/j.cell.2009.05.026 19665970 PMC2726829

[104] Vaiserman A , Krasnienkov D. Telomere length as a marker of biological age: state-of-the-art, open issues, and future perspectives. Front Genet. 2021;11:630186. 10.3389/fgene.2020.630186 33552142 PMC7859450

[105] Shammas MA. Telomeres, lifestyle, cancer, and aging. Curr Opin Clin Nutr Metab Care. 2011;14(1):28–34. 10.1097/MCO.0b013e32834121b1 21102320 PMC3370421

[106] Okamoto K , Seimiya H. Revisiting telomere shortening in cancer. Cells. 2019;8(2):107. 10.3390/cells8020107 30709063 PMC6406355

[107] Timashev LA , Babcock H, Zhuang X, de Lange T. The DDR at telomeres lacking intact shelterin does not require substantial chromatin decompaction. Genes Dev. 2017;31(6):578–589. 10.1101/gad.294108.116 28381412 PMC5393053

[108] Belair CD , Yeager TR, Lopez PM, Reznikoff CA. Telomerase activity: a biomarker of cell proliferation, not malignant transformation. Proc Natl Acad Sci USA. 1997;94(25):13677–13682. 9391085 10.1073/pnas.94.25.13677 PMC28365

[109] Zhang W , Xiong Y, Tao R, Panayi AC, Mi B, Liu G. Emerging insight into the role of circadian clock gene *BMAL1* in cellular senescence. Front Endocrinol (Lausanne). 2022;13:915139. 10.3389/fendo.2022.915139 35733785 PMC9207346

[110] Ahmed R , Reza HM, Shinohara K, Nakahata Y. Cellular senescence and its impact on the circadian clock. J Biochem. 2022;171(5):493–500. 10.1093/jb/mvab115 34668549

[111] Eitsuka T , Nakagawa K, Kato S, et al Modulation of telomerase activity in cancer cells by dietary compounds: a review. Int J Mol Sci. 2018;19(2):478. 10.3390/ijms19020478 29415465 PMC5855700

[112] Ganesan K , Xu B. Telomerase inhibitors from natural products and their anticancer potential. Int J Mol Sci. 2017;19(1):13. 10.3390/ijms19010013 29267203 PMC5795965

[113] Bayat Mokhtari R , Homayouni TS, Baluch N, et al Combination therapy in combating cancer. Oncotarget. 2017;8(23):38022–38043. 10.18632/oncotarget.16723 28410237 PMC5514969

[114] Abubakar IB , Lim KH, Kam TS, Loh HS. Jerantinine B enhances the mitochondria-mediated apoptosis by p53 activation in human glioblastoma cells via a combination with δ-tocotrienol. J Biol Active Prod Nat. 2018;8(1):21–27. 10.1080/22311866.2018.1440253

[115] Chiang AC , Massagué J. Molecular basis of metastasis. N Engl J Med. 2008;359(26):2814–2823. 10.1056/NEJMra0805239 19109576 PMC4189180

[116] Klein CA. The metastasis cascade. Science. 2008;321(5897):1785–1787. 10.1126/science.1164853 18818347

[117] Bakir B , Chiarella AM, Pitarresi JR, Rustgi AK. EMT, MET, plasticity, and tumor metastasis. Trends Cell Biol. 2020;30(10):764–776. 10.1016/j.tcb.2020.07.003 32800658 PMC7647095

[118] Jolly MK , Ware KE, Gilja S, Somarelli JA, Levine H. EMT and MET: necessary or permissive for metastasis? Mol Oncol. 2017;11(7):755–769. 10.1002/1878-0261.12083 28548345 PMC5496498

[119] Shiozawa Y , Nie B, Pienta KJ, Morgan TM, Taichman RS. Cancer stem cells and their role in metastasis. Pharmacol Ther. 2013;138(2):285–293. 10.1016/j.pharmthera.2013.01.014 23384596 PMC3602306

[120] Luo L , Liu P, Zhao K, Zhao W, Zhang X. The immune microenvironment in brain metastases of non-small cell lung cancer. Front Oncol. 2021;11:698844. 10.3389/fonc.2021.698844 34336687 PMC8316686

[121] Neophytou CM , Panagi M, Stylianopoulos T, Papageorgis P. The role of tumor microenvironment in cancer metastasis: molecular mechanisms and therapeutic opportunities. Cancers (Basel). 2021;13(9):2053. 10.3390/cancers13092053 33922795 PMC8122975

[122] Ioannidou E , Moschetta M, Shah S, et al Angiogenesis and anti-angiogenic treatment in prostate cancer: mechanisms of action and molecular targets. Int J Mol Sci. 2021;22(18):9926. 10.3390/ijms22189926 34576107 PMC8472415

[123] Oguntade AS , Al-Amodi F, Alrumayh A, Alobaida M, Bwalya M. Anti-angiogenesis in cancer therapeutics: the magic bullet. J Egypt Natl Canc Inst. 2021;33(1):15. 10.1186/s43046-021-00072-6 34212275 PMC13316910

[124] Bergers G , Benjamin LE. Tumorigenesis and the angiogenic switch. Nat Rev Cancer. 2003;3(6):401–410. 10.1038/nrc1093 12778130

[125] Murciano-Goroff YR , Warner AB, Wolchok JD. The future of cancer immunotherapy: microenvironment-targeting combinations. Cell Res. 2020;30(6):507–519. 10.1038/s41422-020-0337-2 32467593 PMC7264181

[126] De Silva L , Chuah LH, Meganathan P, Fu JY. Tocotrienol and cancer metastasis. Biofactors. 2016;42(2):149–162. 10.1002/biof.1259 26948691

[127] Miyazawa T , Shibata A, Sookwong P, et al Antiangiogenic and anticancer potential of unsaturated vitamin E (tocotrienol). J Nutr Biochem. 2009;20(2):79–86. 10.1016/j.jnutbio.2008.09.003 19071006

[128] Shibata A , Nakagawa K, Sookwong P, et al Tocotrienol inhibits secretion of angiogenic factors from human colorectal adenocarcinoma cells by suppressing hypoxia-inducible factor-1alpha. J Nutr. 2008;138(11):2136–2142. 10.3945/jn.108.093237 18936210

[129] Gonzalez H , Hagerling C, Werb Z. Roles of the immune system in cancer: from tumor initiation to metastatic progression. Genes Dev. 2018;32(19–20):1267–1284. 10.1101/gad.314617.118 30275043 PMC6169832

[130] Candeias SM , Gaipl US. The immune system in cancer prevention, development and therapy. Anticancer Agents Med Chem. 2016;16(1):101–107. 10.2174/1871520615666150824153523 26299661

[131] Corthay A. Does the immune system naturally protect against cancer? Front Immunol. 2014;5:197. 10.3389/fimmu.2014.00197 24860567 PMC4026755

[132] Labani-Motlagh A , Ashja-Mahdavi M, Loskog A. The tumor microenvironment: a milieu hindering and obstructing antitumor immune responses. Front Immunol. 2020;11:940. 10.3389/fimmu.2020.00940 32499786 PMC7243284

[133] Beatty GL , Gladney WL. Immune escape mechanisms as a guide for cancer immunotherapy. Clin Cancer Res. 2015;21(4):687–692. 10.1158/1078-0432.CCR-14-1860 25501578 PMC4334715

[134] Taniguchi K , Karin M. NF-κB, inflammation, immunity and cancer: coming of age. Nat Rev Immunol. 2018;18(5):309–324. 10.1038/nri.2017.142 29379212

[135] Xia L , Tan S, Zhou Y, et al Role of the NFκB-signaling pathway in cancer. Onco Targets Ther. 2018;11:2063–2073. 10.2147/OTT.S161109 29695914 PMC5905465

[136] Hoesel B , Schmid JA. The complexity of NF-κB signaling in inflammation and cancer. Mol Cancer. 2013;12(1):86. 10.1186/1476-4598-12-86 23915189 PMC3750319

[137] Aggarwal BB , Sung B. NF-κB in cancer: a matter of life and death. Cancer Discov. 2011;1(6):469–471. 10.1158/2159-8290.CD-11-0260 22586649 PMC3392037

[138] Khor BH , Tiong HC, Tan SC, et al Effects of tocotrienols supplementation on markers of inflammation and oxidative stress: a systematic review and meta-analysis of randomized controlled trials. PLoS One. 2021;16(7):e0255205. 10.1371/journal.pone.0255205 34297765 PMC8301652

[139] Malavolta M , Pierpaoli E, Giacconi R, et al Anti-inflammatory activity of tocotrienols in age-related pathologies: a SASPected involvement of cellular senescence. Biol Proced Online. 2018;20(1):22. 10.1186/s12575-018-0087-4 30479579 PMC6247629

[140] Wong WY , Ward LC, Fong CW, Yap WN, Brown L. Anti-inflammatory γ- and δ-tocotrienols improve cardiovascular, liver and metabolic function in diet-induced obese rats. Eur J Nutr. 2017;56(1):133–150. 10.1007/s00394-015-1064-1 26446095

[141] Mahalingam D , Radhakrishnan AK, Amom Z, Ibrahim N, Nesaretnam K. Effects of supplementation with tocotrienol-rich fraction on immune response to tetanus toxoid immunization in normal healthy volunteers. Eur J Clin Nutr. 2011;65(1):63–69. 10.1038/ejcn.2010.184 20859299

[142] Radhakrishnan AK , Lee AL, Wong PF, Kaur J, Aung H, Nesaretnam K. Daily supplementation of tocotrienol-rich fraction or alpha-tocopherol did not induce immunomodulatory changes in healthy human volunteers. Br J Nutr. 2009;101(6):810–815. 10.1017/S0007114508039998 18702848

[143] Ramamonjisoa N , Ackerstaff E. Characterization of the tumor microenvironment and tumor–stroma interaction by non-invasive preclinical imaging. Front Oncol. 2017;7:3. 10.3389/fonc.2017.00003 28197395 PMC5281579

[144] Valkenburg KC , de Groot AE, Pienta KJ. Targeting the tumour stroma to improve cancer therapy. Nat Rev Clin Oncol. 2018;15(6):366–381. 10.1038/s41571-018-0007-1 29651130 PMC5960434

[145] Stadler M , Pudelko K, Biermeier A, et al Stromal fibroblasts shape the myeloid phenotype in normal colon and colorectal cancer and induce CD163 and CCL2 expression in macrophages. Cancer Lett. 2021;520:184–200. 10.1016/j.canlet.2021.07.006 34256095

[146] Ratti M , Lampis A, Ghidini M, et al MicroRNAs (miRNAs) and long non-coding RNAs (lncRNAs) as new tools for cancer therapy: first steps from bench to bedside. Target Oncol. 2020;15(3):261–278. 10.1007/s11523-020-00717-x 32451752 PMC7283209

[147] Ling H , Fabbri M, Calin GA. MicroRNAs and other non-coding RNAs as targets for anticancer drug development. Nat Rev Drug Discov. 2013;12(11):847–865. 10.1038/nrd4140 24172333 PMC4548803

[148] Nukala SB , Jousma J, Cho Y, Lee WH, Ong SG. Long non-coding RNAs and microRNAs as crucial regulators in cardio-oncology. Cell Biosci. 2022;12(1):24. 10.1186/s13578-022-00757-y 35246252 PMC8895873

[149] Tornesello ML , Faraonio R, Buonaguro L, et al The role of microRNAs, long non-coding RNAs, and circular RNAs in cervical cancer. Front Oncol. 2020;10:150. 10.3389/fonc.2020.00150 32154165 PMC7044410

[150] Wang C , Ju H, Shen C, Tong Z. miR-429 mediates δ-tocotrienol-induced apoptosis in triple-negative breast cancer cells by targeting XIAP. Int J Clin Exp Med. 2015;8(9):15648–15656. 26629059 PMC4658948

[151] Ji X , Wang Z, Geamanu A, Goja A, Sarkar FH, Gupta SV. Delta-tocotrienol suppresses Notch-1 pathway by upregulating miR-34a in nonsmall cell lung cancer cells. Int J Cancer. 2012;131(11):2668–2677. 10.1002/ijc.27549 22438124 PMC4164155

[152] Ehteram H , Aslanbeigi F, Ghoochani Khorasani E, Tolouee M, Haddad Kashani H. Expression and prognostic significance of stem cell marker CD133 in survival rate of patients with colon cancer. Oncol Ther. 2022;10(2):451–461. 10.1007/s40487-022-00205-4 35980560 PMC9681962

[153] Ren F , Sheng WQ, Du X. CD133: a cancer stem cells marker, is used in colorectal cancers. World J Gastroenterol. 2013;19(17):2603–2611. 10.3748/wjg.v19.i17.2603 23674867 PMC3645378

[154] Ricci-Vitiani L , Lombardi DG, Pilozzi E, et al Identification and expansion of human colon-cancer-initiating cells. Nature. 2007;445(7123):111–115. 10.1038/nature05384 17122771

[155] Luk SU , Yap WN, Chiu YT, et al Gamma-tocotrienol as an effective agent in targeting prostate cancer stem cell-like population. Int J Cancer. 2011;128(9):2182–2191. 10.1002/ijc.25546 20617516

[156] Cheng Y , Ling Z, Li L. The intestinal microbiota and colorectal cancer. Front Immunol. 2020;11:615056. 10.3389/fimmu.2020.615056 33329610 PMC7734048

